# Iron deficiency was not the major cause of anemia in rural women of reproductive age in Sidama zone, southern Ethiopia: A cross-sectional study

**DOI:** 10.1371/journal.pone.0184742

**Published:** 2017-09-12

**Authors:** Tafere Gebreegziabher, Barbara J. Stoecker

**Affiliations:** 1 School of Nutrition, Food Science and Technology, Hawassa University, Hawassa, Ethiopia; 2 Department of Health Sciences, Central Washington University, Ellensburg, Washington, United States of America; 3 Department of Nutritional Sciences, Oklahoma State University, Stillwater, Oklahoma, United States of America; Institut de recherche pour le developpement, FRANCE

## Abstract

**Background:**

Anemia, which has many etiologies, is a moderate/severe public health problem in young children and women of reproductive age in many developing countries. The aim of this study was to investigate prevalence of iron deficiency, anemia, and iron deficiency anemia using multiple biomarkers and to evaluate their association with food insecurity and food consumption patterns in non-pregnant women from a rural area of southern Ethiopia.

**Methods:**

A cross-sectional study was conducted in 202 rural women of reproductive age in southern Ethiopia. Anthropometrics and socio-demographic data were collected. A venipuncture blood sample was analyzed for hemoglobin (Hb) and for biomarkers of iron status. Biomarkers were skewed and were log transformed before analysis. Mean, median, Pearson’s correlations and ordinary least-squares regressions were calculated.

**Results:**

Median (IQR) Hb was 138 (127, 151) g/L. Based on an altitude-adjusted (1708 m) cutoff of 125 g/L for Hb, 21.3% were anemic. Plasma ferritin was <15 μg/L in 18.6% of the women. Only one woman had α-1-acid glycoprotein (AGP) >1.0 g/L; four women (2%) had > 5 mg/L of C-reactive protein (CRP). Of the 43 women who were anemic, 23.3% (10 women) had depleted iron stores based on plasma ferritin. Three of these had elevated soluble transferrin receptors (sTfR). Hemoglobin (Hb) concentration was negatively correlated with sTfR (r = -0.24, p = 0.001), and positively correlated with ferritin (r = 0.17, p = 0.018), plasma iron (r = 0.15, p = 0.046), transferrin saturation (TfS) (r = 0.15, p = 0.04) and body iron (r = 0.14, p = 0.05). Overall prevalence of iron deficiency anemia was only 5%.

**Conclusion:**

Iron deficiency anemia was not prevalent in the study population, despite the fact that anemia would be classified as a moderate public health problem.

## Introduction

Iron deficiency (ID) is a state in which there is insufficient iron to maintain the normal physiological function of tissues [1]. ID without the occurrence of anemia is possible before the concentration of hemoglobin (Hb) falls below the threshold for the specific sex and age group [1]. Iron deficiency is one of the most common micronutrient deficiencies in the world and unlike most nutrient deficiencies, ID is prevalent in both developed and developing nations [2]. Anemia, with or without iron deficiency, is a common disorder worldwide [3].

According to WHO classification, prevalence of anemia of 20 to 39.9% is defined as a moderate public health problem, and 40% and above is defined as a severe public health problem [1]. Estimates of anemia for preschool-age children and women of child-bearing age (both pregnant and non-pregnant women) indicate that the highest proportion, 47.5–67.6%, of individuals affected by anemia are in Africa [1]. Globally, nearly 50% of pregnant women are anemic but this anemia is not always iron deficiency anemia (IDA) [3].

In a survey conducted in 2005, Umeta and colleagues reported that prevalence of anemia in women of reproductive age, in the southern region of Ethiopia was 25% with 20.3% mild anemia and 4.7% moderate anemia; 12.4% of the women were classified as IDA [4]. According to the Ethiopian Demographic and Health Survey (EDHS), national prevalence of anemia has declined from 26.6% in 2005 to 16.6% in 2011. Consistent with the decline in any anemia at the national level, the EDHS reported a decline in the southern region from 22.5% in 2005 to 11.3% in 2011 [5]. Among those, 8.8% had mild anemia, 2.1% had moderate anemia, and 0.4% had severe anemia, which was substantially lower than the national prevalence [5]. Recently, the Ethiopian Public Health Institute (EPHI) has reported that the national prevalence of any anemia in women of reproductive age in 2016 was 17.7% and in the southern region was 13.3% [6].

Hemoglobin values, one of the indicators of iron status, vary with age, sex, state of pregnancy, altitude, and smoking. For these reasons, adjustment is required in population-based surveys when interpreting Hb values [3]. Some studies have used Hb concentration as an indicator of IDA but anemia can be caused by other factors including malaria, parasitic infection, inflammation and nutritional deficiencies in addition to iron deficiency [7].

Ferritin, an iron storage protein, and transferrin receptors which control the entry of iron-bearing transferrin into cells, are major iron biomarkers. The total tissue concentration of ferritin is proportional to the amount detected in the serum and higher serum ferritin indicates the availability of corresponding amounts of storage iron [8]. However, serum ferritin is not reliable as an indicator in the presence of infection because ferritin concentration increases with inflammation as a result of the acute phase response to disease [9].

Anemia is diagnosed when the Hb concentration is lower than the level considered normal for the person’s age, sex and physiological status [10]. The World Health Organization defines anemia as a Hb value below the age-specific 2.5^th^ percentile value in a non-anemic distribution [3]. The accepted cutoff to define anemia in non-pregnant females, above 15 years of age living at sea level is 120 g/L. The altitude adjusted value in our study area is 125 g/L [3]. Adding measures of iron-deficient erythropoiesis such as transferrin iron saturation improves sensitivity for detection of iron deficiency. Estimation of body iron based on the ratio of serum transferrin receptor to serum ferritin enhances the assessment of iron status in populations where inflammation is not prevalent [11, 12].

As mentioned previously, anemia has been identified in the study population. However, information is inadequate on the etiology and related factors for the anemia. The aim of this study was to investigate prevalence of iron deficiency, anemia and iron deficiency anemia using multiple biomarkers in non-pregnant women from rural areas of southern Ethiopia.

## Materials and methods

### Data source and the study population

A cross sectional study was conducted from June to July, 2009, in rural communities of Sidama zone, southern Ethiopia. After announcement throughout the randomly selected villages, 202 non-pregnant women age 18 and older who volunteered to participate were enrolled and were invited to come to the health post for data collection.

### Anthropometry and questionnaire

Weight (kg) and height (cm) of participants were measured to calculate body mass index (BMI = weight (kg)/height (m)^2^). Weight was measured on a solar digital scale (Uniscale, UNICEF, NY, USA) and recorded to the nearest 100 grams. Women wore light clothing and removed shoes and heavy outer wear (e.g. sweaters) before obtaining weight. Height was measured to the nearest 0.1 cm using a single calibrated instrument (Adult Board, Schorr Productions, Olney, MD, USA). Mid upper arm circumference was measured to the nearest 0.1 cm using a plastic measuring tape. Measurement was taken at the mid-point of the upper arm, between the acromion process and the tip of the olecranon. Food consumption pattern was assessed using standardized food frequency questionnaire (FFQ). Anthropometry measurements and FFQ were according to Gibson [13].

A questionnaire was administered to assess socio-economic and demographic characteristics of the women. Additionally, data were collected using the Household Food Insecurity Access Scale (HFIAS) developed by the Food and Nutrition Technical Assistance (FANTA) project of the United States Agency for International Development (USAID) [14]. The nine questions in the HFIAS assess evidence of household concerns ranging from simple worry for food shortage (first question) to experiences of often spending day and night without food during the prior four weeks. Four levels of food insecurity were computed based on the formula provided with HFIAS version 3. The Household Hunger Scale (HHS) also was calculated using the last three questions of the HFIAS. The HHS measures more sustained food shortage/ hunger, and the scores range from 0–6, with higher scores representing increasing severity of household hunger. The cross cultural applications of the HFIAS and HHS were validated in several countries [15], and previous studies in Southern Ethiopia assessed their use and psychometric properties [16, 17].

### Measurement of biomarkers

A fasting morning venipuncture blood sample was collected from each participant using a syringe coated with lithium heparin with a 21 gauge needle (Sarstedt, Inc., Newton, NC, USA). A drop of venous blood from the syringe needle was used for Hb measurement; remaining blood was centrifuged and plasma was separated immediately at the health post where the data collection took place. Plasma was transferred into trace-element-free vials with disposable plastic pipettes. The plasma was transported from the data collection site to Hawassa University in an ice box and stored at -20 ^o^C until transported on dry ice to Oklahoma State University, where 194 samples were available for analysis after removal of eight hemolyzed samples.

#### Hemoglobin

Hemoglobin concentration was measured at the health post with a HemoCue (Hemocue AB, Ängelholm, Sweden) instrument. The Hemocue system is a reliable quantitative method for determining Hb concentrations in field surveys [3]. Correction for altitude (1708 m) of Hb cutoffs was calculated according to the equation recommended by UNICEF/UNU/WHO: Hb (g/dL) = -0.32 x (altitude in meters x 0.0033) + 0.22 x (altitude in meters x 0.0033)^2^ which resulted in a cutoff of 12.5 g/dL or 125 g/L to define anemia at the altitude of our study site [3, 18].

#### Ferritin

Plasma ferritin was analyzed using an immunoradiometric procedure (Ramco Laboratories, Stafford, TX, USA). WHO standard values were used to define the ferritin. Participants were classified as having depleted iron stores if their plasma ferritin concentration was <15μg/L. Because plasma ferritin is elevated in the presence of inflammation, ferritin concentrations have been adjusted based on the recommendations given by Thurnham and others [19, 20]. Hence for the incubation phase (CRP ≥5 mg/L) an adjustment factor of 0.77 and for the late convalescent phase (AGP ≥1 g/L) an adjustment factor of 0.75 have been used. No women were classified as early convalescent stage (elevated CRP and AGP).

#### Soluble transferrin receptors

Soluble transferrin receptor (sTfR) concentration was determined quantitatively based upon a double antibody sandwich method (Ramco Laboratories, Stafford, TX, USA). Concentration of sTfR ≥ 8.3 mg/L was taken to represent a functional iron deficit.

#### Plasma iron, total iron binding capacity (TIBC), and transferrin

Plasma iron was analyzed by inductively coupled plasma mass spectrometer (ICP-MS, Elan 9000, Perkin Elmer, Norwalk, CT, USA) using UTAK serum (Utak Laboratories, Inc., Valencia, CA, USA) for quality control. Total iron binding capacity (TIBC) was analyzed using a BioLis 24i Clinical Analyzer (Carolina Liquid Chemistries Corp., Winston-Salem, NC, USA). Transferrin was quantified by radial immunodiffusion (Kent Laboratories Inc., Bellingham, WA, USA).

#### Transferrin saturation, soluble transferrin receptor and body iron

Transferrin saturation (TfS) was calculated from plasma iron and TIBC (TfS = plasma iron x 100/TIBC). Body iron was calculated using the formula suggested by Cook et al and Mei et al [11, 12] as follows:

$$\mathrm{Body}\;\mathrm{iron}\;\left(\mathrm{mg}/\mathrm{kg}\right)=\text{-}\;\left[\mathrm{lo}g_{10}\left(\mathrm{sTfR}/F\;\mathrm{ratio}\right)–2.8229\right]/0.1207.$$

#### Acute phase proteins

C-reactive protein (CRP) was assessed by ELISA (Helica Biosystems Inc., Fullerton, CA, USA); α-1-acid glycoprotein (AGP) also was assessed by ELISA (GenWay Biotech, Inc., San Diego, CA, USA). As a measure of acute inflammation, CRP concentration was classified as high if it was >5 mg/L. Values greater than 1 g/L for AGP were taken to represent chronic inflammation.

### Statistical analysis

Percentages, frequency distributions, means, standard deviations, medians and interquartile ranges were used as appropriate in describing the socio-economic and demographic characteristics of the respondents, iron biomarkers and inflammation indicators. All data were checked for normal distribution and skewed data were log-transformed before analysis. Pearson’s correlation coefficients were used to examine relations between variables. Confidence intervals were used to describe the variance of odds ratio (OR). Multiple linear regression analysis was used to examine the relation between Hb concentration and independent variables. Collinearity effect was tested using a variance inflation factor (VIF) for all independent variables and the variables that met the criteria (VIF ≤ 4) were entered into the model. All the analyses were performed with SPSS (version 20; IBM Corp. Armonk, NY). Level of significance for correlations was set at p < 0.05.

### Ethical considerations

Ethical approval was obtained from the review boards for Oklahoma State University (OSU), USA, Hawassa University and the Ministry of Science and Technology, Ethiopia. A detailed explanation of the research was given to the community health workers responsible for monitoring the health condition of the rural communities as well as directly to the community women. Subsequently, 202 women, who agreed to participate, returned on a scheduled date and a consent form was read to each woman, signed by finger print and witnessed by the health extension worker before data collection began.

## Results

### Demographic, socioeconomic and anthropometric characteristics of women

Characteristics of the study participants are shown in Table 1. The self-reported mean (SD) age of the participants was 30.8 (7.8) years. Of the participants, 63.2% had no formal education. The mean household size was 6.1 (2.5). The mean number of pregnancies was 4.7 (2.7).

**Table 1:** Anthropometric, demographic and socioeconomic characteristics of women from Sidama zone, southern Ethiopia (n = 202).

Characteristics	Mean (SD), %
Age (years)	30.8 (7.8)
-≤ 25	23.8
-26–35	50.5
-> 35	57.7
School years of women (years)	1.5 (2.5)
-No education	63.2
-1–3 years	15.8
-≥ 4 years	21%
Household size	6.1 (2.5)
-≤ 4	25.2
-5–7	45.1
-≥ 8	29.7
Gravidity	4.7 (2.7)
-≤ 3	36.1
-4–6	41.1
-≥ 7	22.8
Parity	4.2 (2.2)
-≤ 3	41.0
-4–6	44.0
-≥ 7	15.0
BMI (kg m^-2^)	20.0 (2.2)
-≤ 18.5	24.8
-≥ 18.5 - < 25	73.2
-≥ 25	2
MUAC (cm)	24.8 (2.5)
-< 22	9
-≥ 22	91
Size of land (hectare)	0.44 (0.23)
-≤ 0.25	44.1
-> 0.25–0.5	41.6
-> 0.5	14.3
Number of mature enset owned (%)	14.8 (49.7)
-No mature enset	73.5
-1–15	9.5
-> 15–50	11
-≥ 51	6

BMI, body mass index; MUAC, mid-upper arm circumference.

Analysis of body mass index showed that 2.5% of the women were severely thin (BMI < 16), 3% were moderately thin (BMI between 16 and 16.9), and 19.3% had BMI between 17.0 and 18.5. Four women were classified as overweight (BMI > 25) and the remaining 73% had BMI between 18.5 and 24.9 [21].

The mean mid upper arm circumference (MUAC) as presented in Table 1 was 24.8 (2.5) cm. Based on MUAC, two (1%) women were severely wasted (MUAC < 19 cm), 8% were undernourished (MUAC 19 to < 22 cm) and 91% were normal (MUAC ≥ 22 cm) [22].

The average size of farmed land was 0.4 (0.2) hectares and only one participant had as much as 1.5 hectare of land. Mature enset plants (the major staple food in addition to maize as well as an indicator of wealth) were owned by 26% but the number of plants varied from 1 to 500. However, the majority (77%) of owners had less than 50 plants. Eighty five percent of the women owned one or more animals including poultry.

As indicated in Table 2, based on the HFIAS questionnaire administered in June and July, the food shortage season, 44.6% of the participants were severely, 36% were moderately and 4.5% were mildly food insecure within the last month. Calculation of the HHS showed that 23.4% of the participants had moderate and 2.5% had severe household hunger. Among the 36 women who had ferritin level below 15 μg/L, 53% were severely and 30.6% were moderately food insecure. Among the 10 women who had sTfR level 8.3 mg/L and above, eight women were food insecure. There was not a significant difference between food secure (secure and mild insecurity combined) women and those classified as food insecure (moderate and severe combined) in terms of risk for low ferritin (OR = 1.3, 95% CI: 0.48–3.3), elevated sTfR (OR = 1.1, 95% CI: 0.23–5.3) or low Hb (OR = 1.8, 95% CI: 0.69–4.52).

**Table 2:** Percentage distribution of women by level of household food insecurity and hunger scale (n = 202).

Variables	n	Percent
Household food insecurity measures		
Food secure	30	14.9
Mildly food insecure	9	4.5
Moderately food insecure	73	36.0
Severely food insecure	90	44.6
Household hunger measures		
No or little household hunger	149	74.1
Moderate household hunger	47	23.4
Severe household hunger	5	2.5

### Food consumption pattern of women

As shown in Table 3, maize, kidney beans, enset products, kale, cow’s milk, avocado, sweet potato and potato were most commonly consumed foods in the study area. Among these, the most frequently consumed foods were maize with mean of 6.5 (0.8) days per week, enset products with mean of 6.2 (1.3) days per week and kale with mean of 6.2 (1.3) days per week. Of the women 97.5% consumed maize, 94.1% consumed enset products and 95% consumed kale for five and more days a week.

**Table 3:** Frequency of consumption by women of foods commonly available in Sidama zone, southern Ethiopia (n = 202).

Types of foods	Percent	Mean (SD)
Maize (frequency per week)		(0.8)
3–4	2.5	
≥ 5	97.5	
Kidney bean (frequency per week)		5.0 (1.6)
0–2	10.9	
3–4	11.9	
≥ 5	77.2	
Enset products (frequency per week)		6.2 (1.3)
3–4	5.9	
≥ 5	94.1	
Kale (frequency per week)		6.3 (1.3)
3–4	5.0	
≥ 5	95.0	
Cow milk (frequency per week)		2.9 (2.3)
Never	28.2	
1–2	19.8	
3–4	28.7	
≥ 5	23.3	
Avocado (frequency per week)		3.9 (1.7)
Never	7.9	
1–2	16.8	
3–4	34.2	
≥ 5	41.1	
Sweet potato (frequency per week)		2.6 (1.5)
Never	14.9	
1–2	37.6	
3–4	39.6	
≥ 5	7.9	
Potato (frequency per week)		3.6 (1.6)
Never	8.4	
1–2	13.9	
3–4	48.5	
≥ 5	29.2	

### Assessment of iron status biomarkers

To comprehensively assess iron deficiency and anemia, several biomarkers were analyzed and calculated (Table 4). Of the women, 18.6% had depleted iron stores (ferritin < 15 μg/L). Women with plasma iron below 500 μg/L were 27.3%; 5.2% of women had depleted functional iron (sTfR ≥ 8.3 mg/L). Iron deficient erythropoiesis (ferritin < 12 μg/L and plasma iron < 500 μg/L) was observed in 5.4% and 16.9% had early functional iron deficiency (transferrin saturation < 15%). Increased total iron binding capacity was observed in 7.4% which could also indicate depleted iron stores. Tissue iron deficiency (Body iron < 0 mg/kg) was found in only 6.2% of the women.

**Table 4:** Iron status biomarkers and inflammation indicators (CRP and AGP) of rural women from Sidama zone, southern Ethiopia (n = 189–202).

	Percent	Median (IQR)
Plasma iron (μg/L)		(491, 959)
< 500	27.3 (53/194)	
≥ 500	73.7 (143/194)	
Ferritin (μg/L)		(17.8, 43.6)
< 15	18.6 (36/194)	
≥ 15	82.5 (160/194)	
sTfR (mg/L)		3.7 (2.7, 4.9)
< 8.3	(183/193)	
≥ 8.3	5.2 (10/193)	
Transferrin saturation (%)		(18.4, 33.7)
< 15	16.9 (32/189)	
≥ 15	83.1 (157/189)	
TIBC (mg/L)		283 (53, 309.5)
< 3.5	92.6 (175/189)	
≥ 3.5	7.4 (14/189)	
Body iron		0.12 (0.07, 0.17)
< 0 (mg/kg)	6.2 (12/193)	
> 0 (mg/kg)	93.8 (181/193)	
CRP (mg/L)		0.24 (0.14, 0.58)
< 5.0	98.0 (190/194)	
≥ 5.0	2.0 (4/194)	
AGP (g/L)		0.32 (0.24, 0.43)
≤ 1.0	(193/194)	
> 1.0	0.5 (1/194)	
Hemoglobin (g/L)		138 (127, 151)
< 125	(43/202)	
≥ 125	78.7 (159/202)	

sTfR, Serum transferrin receptor; TIBC, total iron binding capacity; CRP, C-reactive protein; AGP, α-1-acid glycoprotein. Hb and plasma iron were measured on 202 samples. Other analyses were limited by sample volume or hemolysis.

Median (IQR; 25, 75) Hb concentration was 138 (127, 151)g/L. The altitude specific Hb concentration assessment indicated that 21.3% of the women were anemic with Hb below 125 g/L (adjusted for altitude of 1708 m). However out of 43 women with Hb concentration < 125 g/L only 10 women (23.3%) had depleted body iron stores (ferritin concentration below 15 μg/L). Moreover, only 10 women who had Hb concentration < 125 g/L had low transferrin saturation, only three had elevated sTfr and only 16 had low plasma iron concentration. Overall, IDA based on Hb concentration and plasma ferritin was only 5%.

Correlation coefficients of iron biomarkers revealed that Hb concentration was negatively correlated with sTfR (r = -0.24, p = 0.001) and positively correlated with ferritin (r = 0.17, p = 0.018), plasma iron (r = 0.15, p = 0.046), TfS (r = 0.15, p = 0.04) and body iron (r = 0.14, p = 0.05). Soluble transferrin receptor concentration was negatively correlated with plasma iron (r = -0.17, p = 0.022) and ferritin (r = -0.18, p = 0.013). These significant correlations support the presence of iron deficiency and IDA.

In Table 5, results from a best-fitting multiple linear regression model with twelve predictor variables for Hb concentration are presented. The variables included were socio-demographic, HFIAS, food frequency and iron biomarkers. Among these, three variables were significant predictors.

**Table 5:** Ordinary least-squares regression analysis for variables predicting hemoglobin concentration in women of reproductive age in Sidama zone, southern Ethiopia.

Variable	β	95% (CI)	p
Constant	15.9	11.7, 20.18	
Age of women	0.05	-0.03, 0.06	0.58
Body mass index	-0.09	-0.22, 0.05	0.19
Gravidity	0.02	-0.15, 0.17	0.89
Household size	-0.18	-0.27, -0.01	**0.033**
School years attended	-0.03	-0.14, 0.09	0.73
Land size	0.02	-1.24, 1.62	0.79
HFIAS	-0.15	-0.09, -0.001	0.06
Frequency of enset products consumption	0.12	0.02, 0.41	0.08
Frequency of maize consumption	-0.18	-0.82, -0.10	**0.013**
Frequency of kale consumption	0.10	-0.07, 0.38	0.17
Transferrin saturation	0.16	0.00, 0.04	0.053
Body iron	0.18	0.69, 7.26	**0.018**
R^2^	18.0		

HFIAS, Household Food Insecurity Access Scale. The dependent (hemoglobin concentration) and all independent variables are continuous. Coding: Age of women (18–52 years), body mass index (15.4–26.1 kg/m^2^), Gravidity (0–14), household size (0–12), school years attended (0–12), land size (0–1.5 ha), HFIAS scores ranging from (0 [no food insecurity] to 27 [the most food insecurity], weekly enset products consumption (3–7), weekly maize consumption (3–7), weekly kale consumption (3–7), transferrin saturation (4.0–92%), and body iron (-15.8 to 19.2 mg/kg).

Household size and frequency of maize consumption were negative predictors and body iron was a positive predictor. A one unit increase in household size was associated with 0.18 g/L decrease in Hb concentration. Each unit increase in frequency of maize consumption resulted in a decrease of 0.18 g/L in Hb. Conversely, an increase of 1 mg/kg body iron was associated with a 0.17 g/L increase of Hb concentration. Hemoglobin concentration tended to be negatively associated with HFIAS (p = 0.056) and positively associated with TfS (0.053).

## Discussion

The prevalence of all-causes of anemia in the study population was 21.3%, which is lower than anemia prevalence in non-pregnant women in many other countries in Africa (47.5%) and globally (30.2%) [1]. Furthermore, iron deficiency anemia (IDA) was only 5%. Abebe and colleagues have reported similar results in communities near our study area in which 23.5% of the pregnant women were anemic and IDA was 8.7% [23]. Umeta and colleagues found 25% anemia in women of reproductive age in the southern region [4]. Other recently published articles also have reported that anemia is a moderate, but not severe public health problem in both women and children in several regions of Ethiopia [24–27].

Although there is some agreement that anemia in Ethiopia is lower than global rates, other studies reported that IDA is still a problem. Haidar and Pobocik found 18.0% IDA, based on Hb and serum ferritin concentration, which classifies IDA as a moderate public health problem according to WHO standards [1, 28]. Consistent with this Umeta and colleagues reported 17% IDA in women of reproductive age nationally [4]. In our study, of the 43 participants who were anemic, only 10 women (5% of the total study participants) were actually classified as IDA based on plasma ferritin concentrations. The rest of the anemia could be as a result of malaria, inflammation or other essential nutrient deficiencies [5, 9].

Further analysis of the data using ordinary least-squares regression showed that Hb concentration was associated with three key predictors with similar degrees of strength. These predictors, as reported in Table 5, were frequency of maize consumption, body iron status and household size. Previous local studies [29], have reported a similar set of predictors of Hb concentration. That frequency of maize consumption was a negative predictor of Hb in this study is particularly interesting because plant-based diets are high in phytate and phytate inhibits mineral absorption [30]. A previous study in the area identified the high phytate: iron ratio in locally produced maize [31].

Due to the fact that the body depends on diet as a source of iron, IDA is often seen in localities where food insecurity is prevalent [17, 32]. However food insecurity did not seem to be a major contributor to anemia or iron deficiency in this study. A majority of our study participants were highly food insecure with 45% severely and 36% moderately food insecure which is consistent with a study of 1094 randomly sampled households in a similar study area which found 48% severely and 28% moderately food insecure [17]. In the present study women who were food insecure were not at a significantly greater risk for low serum ferritin or elevated soluble transferrin receptors. However, the small sample size in this study might explain the lack of significant effect of food insecurity on anemia and iron deficiency.

According to WHO (2011), iron deficiency is not classified as prevalent in populations where percentage of serum ferritin < 15 μg/L is lower than 20% and percentage of sTfR values above cut-offs is lower than 10% [33]. Thus iron deficiency would not be classified as a public health problem in our study participants.

There are reports indicating that the Ethiopian diet is high in iron [34]. Although the food consumption behavior varies from region to region, the diet is mostly cereals [35]. Hofvander suggested that the high iron intake is due not only to the food but also to contaminant iron from sources such as dirt acquired during threshing or from cooking vessels [36]. Weighed food records for 58 women in our study area showed median intake of iron was 28.8 (23.5, 35.0) mg per day [37].

Yigzaw and colleagues have indicated that fermentation enhances nutritive value of cereals by increasing availability of proteins and improving amino acid content. Fermentation also reduces nutrient inhibitor compounds such as phytates [38]. Consistent with this, Gibson and colleagues suggested that absorption of non-heme iron may be facilitated by consumption of fermented enset products (major staple foods), with maize by the study population [39]. Furthermore enset, a staple food in the region has shown high variability in iron content of 0.7 to 6.2 mg iron per 100 g among its different fermented food products [31].

Several studies have determined prevalence of anemia in Ethiopia, but few have assessed multiple biomarkers of iron deficiency. Because some iron status biomarkers are influenced by inflammation, determining prevalence of iron deficiency and its related consequences could be problematic [9]. Therefore assessing multiple biomarkers, particularly serum ferritin, soluble transferrin receptor, Hb concentration and inflammation markers is of great importance [9].

A limitation of this study is the convenience sampling which may not represent the population. Also, food intakes collected by recall are subject to bias but the results of the FFQ from this study are similar to a study in the area with weighed records [23].

## Conclusion

Although anemia was found in 21.3% of the women tested in this study, IDA itself was only 5% when serum ferritin < 15 μg/L was included for classifying IDA. Iron status assessed using multiple additional biomarkers also supported a relatively low level of actual iron deficiency. Because these women only rarely consumed animal foods, further study is needed to investigate the dietary bioavailability of iron of the study population as well as other contributors to anemia.
