# Correction: Iron deficiency was not the major cause of anemia in rural women of reproductive age in Sidama zone, southern Ethiopia: A cross-sectional study

**DOI:** 10.1371/journal.pone.0189553

**Published:** 2017-12-07

**Authors:** The PLOS ONE Staff

There is an error in Table 3. The “Mean” value for “Maize (frequency per week)” is missing. Please see the correct Table 3 here.

**Table 3:** Frequency of consumption by women of foods commonly available in Sidama zone, southern Ethiopia (n = 202).

Types of foods	Percent	Mean (SD)
Maize (frequency per week)		6.5 (0.8)
3–4	2.5	
≥ 5	97.5	
Kidney bean (frequency per week)		5.0 (1.6)
0–2	10.9	
3–4	11.9	
≥ 5	77.2	
Enset products (frequency per week)		6.2 (1.3)
3–4	5.9	
≥ 5	94.1	
Kale (frequency per week)		6.3 (1.3)
3–4	5.0	
≥ 5	95.0	
Cow milk (frequency per week)		2.9 (2.3)
Never	28.2	
1–2	19.8	
3–4	28.7	
≥ 5	23.3	
Avocado (frequency per week)		3.9 (1.7)
Never	7.9	
1–2	16.8	
3–4	34.2	
≥ 5	41.1	
Sweet potato (frequency per week)		2.6 (1.5)
Never	14.9	
1–2	37.6	
3–4	39.6	
≥ 5	7.9	
Potato (frequency per week)		3.6 (1.6)
Never	8.4	
1–2	13.9	
3–4	48.5	
≥ 5	29.2	

There are a number of errors in Table 4. The “Median” values for “Plasma iron (μg/L)”, “Ferritin (μg/L)”, and “Transferrin saturation (%)” are missing. The “Percent” values for “< 8.3” under “sTfR (mg/L)”, “≤ 1.0” under “AGP (g/L)”, and “< 125” under “Hemoglobin (g/L)” are missing. Please see the correct Table 4 here.

**Table 4:** Iron status biomarkers and inflammation indicators (CRP and AGP) of rural women from Sidama zone, southern Ethiopia (n = 189–202).

	Percent	Median (IQR)
Plasma iron (μg/L)		709 (491, 959)
< 500	27.3 (53/194)	
≥ 500	73.7 (143/194)	
Ferritin (μg/L)		28.5 (17.8, 43.6)
< 15	18.6 (36/194)	
≥ 15	82.5 (160/194)	
sTfR (mg/L)		3.7 (2.7, 4.9)
< 8.3	94.8 (183/193)	
≥ 8.3	5.2 (10/193)	
Transferrin saturation (%)		24.7 (18.4, 33.7)
< 15	16.9 (32/189)	
≥ 15	83.1 (157/189)	
TIBC (mg/L)		283 (53, 309.5)
< 3.5	92.6 (175/189)	
≥ 3.5	7.4 (14/189)	
Body iron		0.12 (0.07, 0.17)
< 0 (mg/kg)	6.2 (12/193)	
> 0 (mg/kg)	93.8 (181/193)	
CRP (mg/L)		0.24 (0.14, 0.58)
< 5.0	98.0 (190/194)	
≥ 5.0	2.0 (4/194)	
AGP (g/L)		0.32 (0.24, 0.43)
≤ 1.0	99.5 (193/194)	
> 1.0	0.5 (1/194)	
Hemoglobin (g/L)		138 (127, 151)
< 125	21.3 (43/202)	
≥ 125	78.7 (159/202)	

sTfR, Serum transferrin receptor; TIBC, total iron binding capacity; CRP, C-reactive

protein; AGP, α-1-acid glycoprotein. Hb and plasma iron were measured on 202 samples.

Other analyses were limited by sample volume or hemolysis.

The publisher apologizes for the errors.
